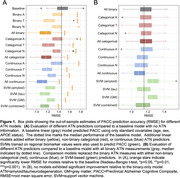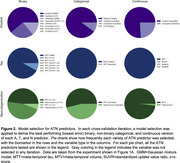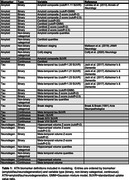# Evaluation of multiple ATN biomarker definitions for prediction of cognitive impairment

**DOI:** 10.1002/alz.092507

**Published:** 2025-01-09

**Authors:** Tom Earnest, Braden Yang, Deydeep Kothapalli, Aristeidis Sotiras

**Affiliations:** ^1^ Mallinckrodt Institute of Radiology, Washington University School of Medicine in St Louis, St Louis, MO USA; ^2^ Institute for Informatics, Washington University, St. Louis, MO USA

## Abstract

**Background:**

Application of the amyloid/tau/neurodegeneration (ATN) framework is varied, with some research relying on binary assessment of biomarkers and some using continuous or multidimensional measures. There are few investigations which directly evaluate how differing operationalizations of ATN affect prediction of cognitive impairment.

**Method:**

We selected 473 individuals from ADNI who received PET imaging for amyloid‐beta (AV‐45) and tau (flortaucipir), as well as volumetric MRI. We evaluated a wide range of methods for deriving ATN biomarker assessments, spanning continuous, binary, and non‐binary categorical (i.e., staging models or quantiles of continuous values) measures (Table 1). We trained linear regression models to predict Preclinical Alzheimer Cognitive Composite (PACC) scores, combining different variations of ATN measures as predictors. We additionally used support vector machines (SVMs) to derive multivariate ATN measures; these models used regional PET uptakes or gray matter volumes (68 FreeSurfer regions) to predict PACC. A first set of experiments tested how addition of ATN improved prediction over a baseline model with just covariates (age, sex, APOE status), while a second set of experiments tested how models including non‐binary or SVM‐based ATN measures compared to ones using only binary measures. We used a repeated, nested, and cross‐validated design to evaluate the out‐of‐sample model performance as quantified by the root mean square error. Error estimates were compared using bias‐corrected t‐tests.

**Result:**

Compared to models with standard covariates, addition of any ATN measure resulted in an increase in accuracy for predicting cognitive ability (Figure 1A). Benefits were largest for models which included tau or all three ATN assessments. SVM‐based models also outperformed baseline models. However, when compared to models using all‐binary ATN measures, addition of categorical, continuous, or SVM‐based ATN measures did not significantly improve prediction accuracy in any combination (Figure 1B). Feature importance analysis indicated that some ATN definitions consistently outperformed others (Figure 2).

**Conclusion:**

Binary measures of ATN may be sufficient for prediction of cross‐sectional cognitive impairment. Further work is required to evaluate if these results hold in specific subpopulations (e.g., preclinical AD) or for predicting other measures of interest (e.g., longitudinal cognitive decline).